# Superficial Mucocele: A Rare Presentation

**DOI:** 10.7759/cureus.18038

**Published:** 2021-09-17

**Authors:** Divyambika C Venugopal, Aravind Warrier S, Elengkumaran S., Thamizhchelvan H, Preethi Ramesh

**Affiliations:** 1 Oral Medicine and Radiology, Sri Ramachandra Institute of Higher Education and Research, Chennai, IND; 2 Oral and Maxillofacial Surgery, Sri Ramachandra Institute of Higher Education and Research, Chennai, IND; 3 Oral and Maxillofacial Pathology, Sri Ramachandra Institute of Higher Education and Research, Chennai, IND

**Keywords:** recurrence, mucocele, minor salivary gland, hard palate, excision

## Abstract

Superficial mucoceles are benign, small, translucent vesicles occurring in any part of the oral cavity, due to extravasation of saliva due to ruptured minor salivary gland ducts. This distinct entity presents as single or multiple asymptomatic vesicles. The etiology is unclear; however, these are not associated with a history of trauma, unlike the conventional mucoceles. These lesions tend to be recurrent and are occasionally associated with discomfort to the patients. Since they clinically mimic various vesiculobullous lesions such as pemphigoid, bullous lichen planus, or herpes virus infection, they are often misdiagnosed. Asymptomatic superficial mucoceles or lesions in multiple locations do not require treatment. Nevertheless, the treatment for lesions causing discomfort includes surgical excision, cryosurgery, and carbon dioxide (CO_2_) laser. This case report describes a rare presentation of superficial mucocele along the midline of the hard palate in a 30-year-old male patient, which was histopathologically confirmed post-surgical excision; the patient is currently disease free with no recurrence for six months.

## Introduction

Superficial mucoceles are reported as translucent vesicles occurring in any part of the oral cavity where there is presence of minor salivary glands [1]. Eveson, in 1988, first reported superficial mucoceles as tense translucent vesicle which tend to rupture and recur, causing discomfort to patients [2]. Case reports with superficial mucoceles have been reported previously in different sites of the oral cavity such as soft palate, lower lip, upper and lower labial mucosa, ventral surface of the tongue, and floor of the mouth [1,3]. Although the occurrence of such lesions are more common in a single location, simultaneous presentation of similar lesions in multiple sites of the oral cavity have been reported previously [1,4]. The present case describes the clinical and histological presentation of superficial mucocele involving the hard palate in a male patient.

## Case presentation

**Figure 1:**
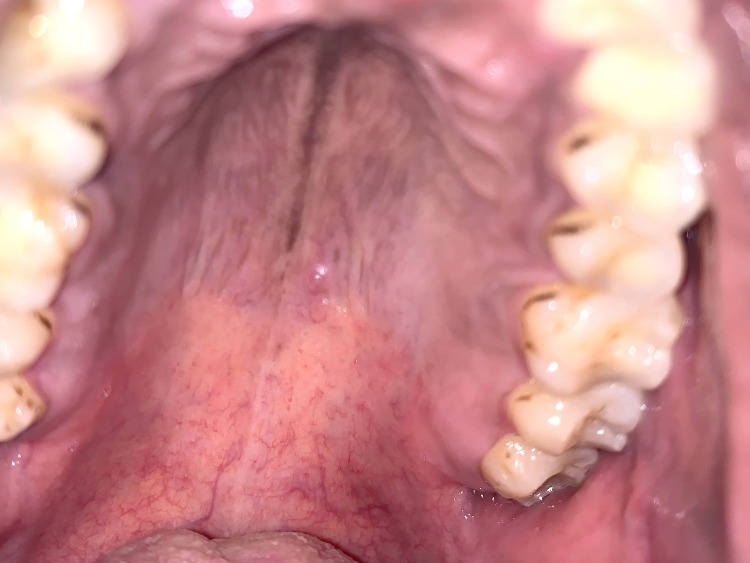
Clinical photograph showing two dome-shaped, 3 x 3 mm vesicles along the midline of the hard palate.

A 30-year-old male patient presented to the oral medicine outpatient department with a chief complaint of blisters in the roof of the mouth for one month. He claimed that the swelling ruptured upon slight trauma with history of recurrence subsequently. Intraoral soft tissue examination revealed evidence of two dome shaped, vesicles of size 3 x 3 mm in the left posterior region of the hard palate, along the midline. On palpation, the vesicles were firm in consistency, tender with no evidence of any discharge (Figure 1).

**Figure 2:**
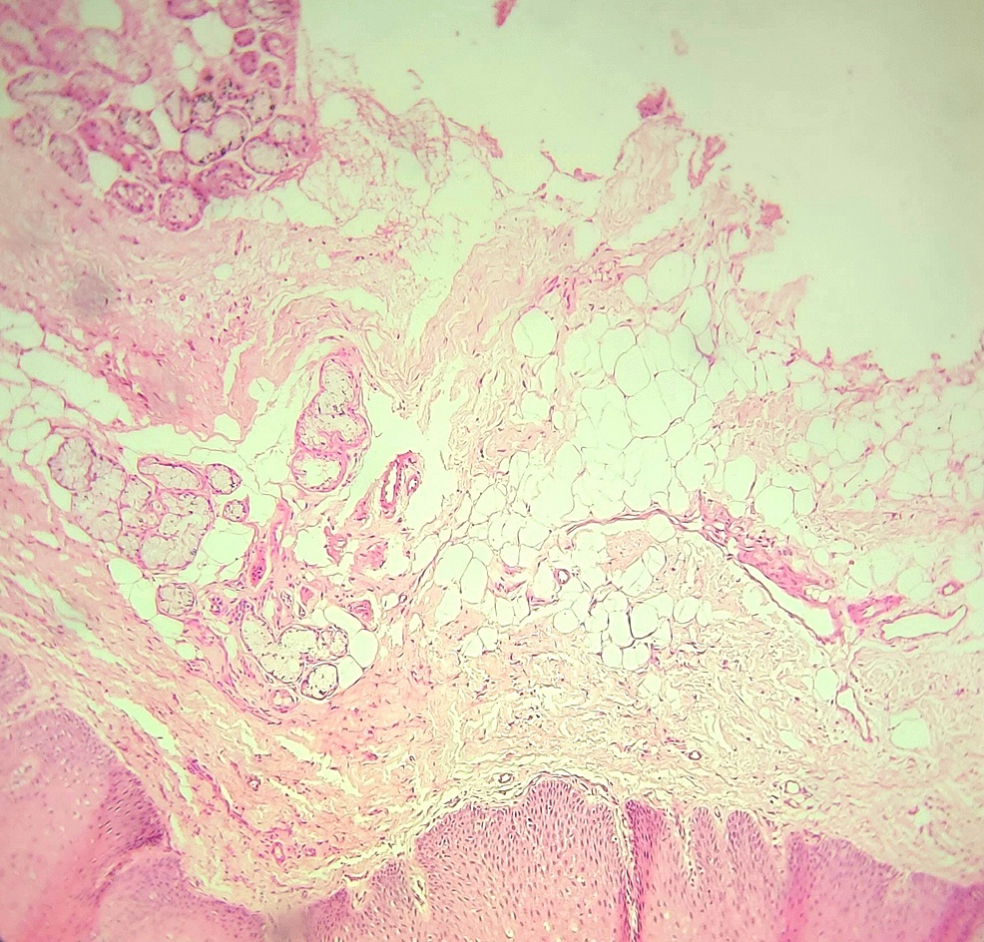
Hematoxylin and eosin (H&E) stain (10x) showing extravasated mucin-containing mucinophages from adjacent minor salivary glands.

No tooth related abnormality was detected clinically; intraoral periapical radiograph and occlusal radiograph did not reveal any pathology. Correlating the history and clinical features, a provisional diagnosis of superficial mucocele in the hard palate was given. Differential diagnosis of vesiculobullous lesions such as mucous membrane pemphigoid, bullous lichen planus, and herpes infection was considered. The hemoglobin levels were with in normal limits and excisional biopsy of the swelling was performed. The histopathology revealed the presence of extravasated mucin containing mucinophages from adjacent minor salivary glands, extravasated RBCs and few inflammatory cells, with peripheral para-keratinized stratified squamous epithelium, suggestive of mucocele (Figure 2).

The healing was satisfactory post-surgical excision and there was no recurrence at six months follow up.

## Discussion

Superficial mucoceles are distinct entities representing sub-epithelial extravasation of sialomucin at the interface of the epithelium and connective tissue [1]. It has been suggested that these lesions could be formed due to ductal rupture secondary to increased pressure caused by the mucous plugs in the intraepithelial squamous-cell-lined portion of the duct. Clinically, these lesions appear as translucent vesicles of few millimetres to up to a centimetre in size, occurring in various sites of the oral cavity which harbour minor salivary glands [3,4]. Most of the cases in the literature have been reported in women, thus suggesting a higher predilection for women, however, our present was a male, which may add to the existing literature on cases reported in males [1-4]. In the present case, the patient was 30 years old; however, superficial mucoceles are more frequently seen in patients of age more than 30 years, unlike mucoceles which are encountered in younger individuals [2,5]. Brooks et al. reported that, in the database created using clinical information for 39 patients of superficial mucocele, only nine patients were male with mean age reported to be 48.9 years [6]. The clinical appearance of these lesions are translucent vesicles, often resembling other vesiculobullous lesions, and generally, rupture spontaneously upon slight trauma with a tendency for recurrence [4]. The classical clinical appearance of asymptomatic, tense, translucent vesicle, ruled out the other probable differential diagnosis such as mucous membrane pemphigoid, bullous lichen planus, and herpes infection [1,2]. Histopathological presentation includes dome-shaped, thin epithelial roof with extravasated mucin and admixed with granulation tissue, inflammatory cells, and foamy muciphages with mild inflammatory cell infiltrate of the lamina propria in the floor [5].

It has been suggested that superficial mucoceles are self-limiting; no biopsy or treatment is necessary, especially for asymptomatic cases [2,3,6]. Xu et al. kept the patients under observation since they were asymptomatic and with numerous lesions in multiple sites [4]. Surgical excision is the preferred treatment, however, scar formation at the excision site and incomplete removal of the adjoining minor salivary glands leading to recurrence are common drawbacks associated with surgical excision [4]. Cryotherapy and laser ablation using carbon dioxide (CO_2_) [7], Neodymium: Yttrium-Aluminium Garnet laser therapy [8] have been tried successfully for alleviating patient discomfort and prevent a recurrence. A previously reported case of multiple superficial mucoceles in allergic stomatitis was successfully treated with betamethasone mouthwash with no history of recurrence for 18 months [1]. A case of multiple superficial mouth mucoceles was treated using oral gamma-linolenic acid; however, relapse occurred once the treatment was suspended [9]. In the present case, the patient underwent surgical excision since he was symptomatic. He is currently disease free with no recurrence for six months.

## Conclusions

Superficial mucoceles are a relatively rare and distinct entity. Identification of such benign lesions by clinicians is usually through proper history taking and thorough intra oral examination, which helps to arrive at an accurate diagnosis. Though such lesions pose a diagnostic and therapeutic challenge to the clinician because of close resemblance of clinical presentation of lesions with different etiologies such as reactional inflammatory processes, infections, immune and iatrogenic responses, systematic clinical examination aided with histopathological evaluation will avoid misdiagnosis and enable better management of patients with superficial mucoceles.
